# Pre‐Sport Cardiac Evaluation in Children: Detection of Risks Other Than Sudden Cardiac Death

**DOI:** 10.1111/anec.70197

**Published:** 2026-06-05

**Authors:** Mehmet Öncül, Abdulgani Gülyüz

**Affiliations:** ^1^ Pediatric Cardiology Malatya Training and Research Hospital Malatya Turkey; ^2^ Faculty of Medicine, Child Health and Diseases Turgut Ozal University Malatya Turkey

**Keywords:** child, echocardiography, electrocardiography, heart, pre‐sports evaluation, sport

## Abstract

**Objective:**

Sudden deaths may occur in children and adolescents due to unexpected cardiac problems during sports. This study aimed to screen for abnormalities that may lead to future morbidity in addition to the risk of sudden death and to investigate the importance of electrocardiography and echocardiography in detecting cardiac problems before sports license.

**Method:**

Pediatric patients who visited the Pediatric Cardiology outpatient clinic of Malatya Training and Research Hospital between April 2022 and April 2024 to obtain a sports license report and who did not have any known heart disease before were included. Data were analyzed using SPSS 22.0.

**Results:**

A total of 2432 patients were admitted to our hospital for pre‐sport evaluation within 2 years. In 2089 of those, there were no known heart problems. Various heart problems were detected in 195 (9.3%) of the 2089 cases. In our study, 169 (8.1%) patients had echocardiography (ECHO) and 33 (1.7%) had electrocardiography (ECG) abnormality. Eight patients (0.3%) showed pathological findings on both ECHO and ECG. In addition, elevated arterial blood pressure was detected in three (0.14%). Of the cases with cardiac abnormality, 110 (56%) were male and 85 (46%) were female, and the average age was 12 (5–18).

**Conclusion:**

Our study shows that approximately one out of every 10 patients in the population may have cardiological problems and 2.3% of these patients have major cardiac problems. Therefore, we recommend that all pediatric patients undergo a detailed cardiac evaluation at least once in the early stages of their lives.

## Graphical abstract

In children who want to play sports, is a cardiac evaluation necessary only for sudden cardiac death?

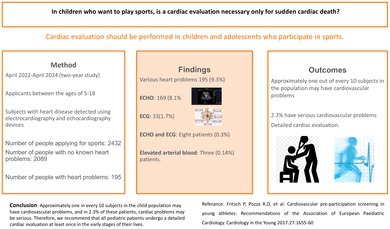

## 1 Introduction

The purpose of pre‐participation evaluation in sports is to detect and prevent abnormalities that may cause sudden death. It has been suggested that the sports‐related mortality rate varies between 0.5 and 13.5 per 100,000 (Germann and Perron 2005; Malhotra et al. 2018).

How to perform cardiovascular evaluations before participation in sports and how effective these evaluations have been the subject of debate for many years. Many findings encountered in individuals who participate in sports and appear abnormal may be normal. In addition, major cardiac problems may be encountered in asymptomatic athletes. Therefore, the main purpose of pre‐participation examinations in sports is to reveal underlying and hidden cardiovascular abnormalities that may cause sudden cardiac death (Thompson 2007).

There is consensus among most international medical and sports organizations that some form of pre‐participation screening should be performed in asymptomatic young athletes to identify abnormalities that may cause disease progression or sudden death. All recommendations included family and personal history questionnaires and physical examination. The necessity of resting 12‐lead electrocardiography (ECG) remains an important scientific debate between the American Heart Association (AHA) and European Society of Cardiology (ESC) recommendations (Harrast and Finnoff 2021; Corrado et al. 2006).

There is consensus among most international medical and sports organizations that some form of pre‐participation screening should be performed in asymptomatic young athletes to identify abnormalities that may cause disease progression or sudden death.

The introduction of mandatory health screening, including ECG as part of the protocol, prior to sports participation has reduced the incidence of sudden cardiac death (Corrado et al. 2006). The strategies for implementing screening programs depend on some specific socioeconomic and cultural conditions and medical system practices specific to different countries.

In these evaluations, the American Heart Association states that a routine resting ECG is not necessary for cost (Maron et al. 2009). According to the European Society of Cardiology, routine ECG application was included (Corrado, Pelliccia, et al. 2005). While the International Olympic Committee and European Society of Cardiology advocate ECG screening, the American Heart Association and American College of Cardiology do not currently recommend an ECG or ECHO for mass screening purposes (Corrado, Pelliccia, et al. 2005; Bille et al. 2006; Myerburg and Vetter 2007; Chaitman 2007).

The American Heart Association recommends that athletes undergo a cardiovascular screening that includes an individual and family history and a physical examination every 2–4 years before and after participation in sports, focusing on cardiovascular problems that may be associated with exercise (Thompson 2007). For this purpose, the American Heart Academy revised the guidelines and developed a questionnaire form with 14 parameters (Maron et al. 2014).

These rare deaths, which occur during or immediately after sports activities, are of concern to parents of children, teachers, and academies organizing sports activities. Unsuspected cardiovascular diseases usually cause these deaths and have been reported with increasing frequency in both the United States and Europe (Maron 2005). This study aims to screen for abnormalities that may lead to future morbidity, in addition to the risk of sudden death. Therefore, most investigations are aimed at detecting any cardiac or non‐cardiac causes of sudden death. The real benefit of such screening is not only the identification of athletes with hereditary heart disease but also the identification of risk in many family members, which may save many lives. Therefore, we aimed to evaluate the effectiveness of ECG and ECHO in detecting structural and/or functional heart problems that may cause sudden death and/or increase mortality and morbidity in later life.

## 2 Methods

This study was conducted retrospectively on the system regarding patients who applied for sports by scanning data using the ICD Z02.5 code. The study data were obtained by scanning the files registered in the hospital's data system. This study was conducted in accordance with the Helsinki Declaration, with the consent of the patients or their relatives. Approval number 223,005 was obtained from the Turgut Özal University Clinical Research Ethics Committee on May 13, 2024.

All patients aged 5–18 who applied to Malatya Training and Research Hospital to start sports between April 1, 2022 and April 1, 2024 were retrospectively evaluated.

Since a health report is routinely requested from primary health care facilities before starting sports as a matter of national policy, these cases were children who applied and were referred to sports clubs, sports course centers, or academies they went to and were directed to do sports.

In our hospital, all patients who apply for pre‐sports evaluation are given the necessary sports reports after history, physical examination, ECG, and ECO evaluation.

### 2.1 Electrocardiographic Evaluation

A 12‐lead ECG device was obtained using an İocare 2019 Digital Electrocardiograph İE 12A model device. In electrocardiography, heart rate, the PR interval, QT and corrected QTc, and intraventricular conduction abnormalities were recorded.

### 2.2 Echocardiographic Evaluation

Evaluated using Philips Affinity 50 2021 model equipment. Structural and functional data were recorded using 2‐dimensional, M‐mode, color, and flow Doppler.

Mitral valve regurgitation (MR) not observed in every beat was considered physiological regurgitation. These patients were not evaluated as having MR. Flattening of the anterior leaflet of the mitral valve was also not considered MVP. Mitral valve prolapse was defined as a displacement of at least 2 mm.

Mitral valve regurgitation was defined according to jet length on color Doppler. Jet length ≤ 1.5 cm was mild, 1.5–2.9 cm moderate, 3–4.4 cm moderate–severe, and ≥ 4.5 cm considered severe MR.

The diagnosis of Acute Rheumatic Fever was made based on a history of previous group A B hemolytic streptococcal infection or laboratory evidence, anterior mitral valve tip thickness (5 mm), history of arthritis, and other laboratory findings.

Tricuspid valve regurgitation was evaluated in patients with jet length over 2.5 m/s.

The diseases that are accepted as severe heart problems and recommended to be banned from sports or which may cause sudden death are hypertrophic cardiomyopathy, MVP, aortic valve stenosis, ventricular arrhythmias, supraventricular arrhythmias, WPW, channelopathies, arterial hypertension, aortic rupture, dilated cardiomyopathy, arrhythmogenic right ventricle, and coronary artery diseases.

### 2.3 Patient Selection

#### 2.3.1 Inclusion Criteria

Patients were selected from among those under 18 years of age who had been registered for sports. Patients who were registered for sports were identified using the ICD Z02.5 code in the patient files. The 2089 patients who underwent ECG and ECHO evaluations and had never previously visited a cardiology outpatient clinic within the national health system were included in the study. Among the patients with pathological findings on ECG, those who had undergone rhythm Holter and exercise testing were included in the study.

#### 2.3.2 Exclusion Criteria

Patients with any previous cardiac abnormality or repeated applications were excluded from the study. Patients who were given a sports report but had missing ECO and ECG data were excluded from this study. Patients with minimal valve regurgitation on echocardiography were excluded from this study. Patients with mitral valve flattening only in the anterior leaflet were excluded from this study.

### 2.4 Statistical Evaluation

Statistical analyses were performed using SPSS 22.0. Normally distributed values were expressed as mean ± standard deviation, while non‐normally distributed values were expressed as median (minimum and maximum) and percentage. To analyze the differences between categorical variables, Pearson's chi‐square and Fisher's exact tests were used for those with normal distribution. Mann Whitney U test was used for those without normal distribution. Statistical significance was set at ≤ 0.05.

## 3 Results

A total of 2432 patients were admitted to our hospital for a pre‐sports evaluation within 2 years. In 2089 of these patients, no heart problems were known to exist before. Of these 2089 patients, 195 (9.3%) had various heart problems. In our study, 169 (8.1%) patients had echocardiographic and 33 (1.7%) patients had electrocardiographic abnormality. Eight patients (0.3%) showed pathological findings on both ECHO and ECG. In addition, three patients (0.14%) had high blood pressure despite normal ECHO and ECG results. Of the patients with heart problems, 110 (56%) were male and 85 (46%) were female, with an average age of 12 (5–18). The demographic data of the patients is shown in Table 1.

**TABLE 1:** Demographic data of cases.

Data	Female (*n*: 85) Average/SD	Male (*n*: 110) Average/SD	*p*
Age	12.5/3.2	11.5/3.0	0.999
Height (cm)	146.5/15.0	151.7/18.3	0.034
Weight (kg)	43.2/13.9	49.0/17.8	0.014
Heart Rate	87.2/7.2	82.9/12.1	0.003
BP systolic	106.8/8.9	109.5/9.2	0.038
BP diastolic	64.5/5.3	65.6/5.8	0.155

Abbreviation: BP, blood pressure arterial.

Mitral valve regurgitation of varying degrees was detected in 85 (50%) patients with pathological findings on echocardiography. Of these patients, 47 (55%) had mild MR, 22 (26%) had moderate MR, and 16 (19%) had moderate‐to‐severe MR.

In addition, mitral valve prolapse (MVP) was found in 24 (14%) patients, secundum atrial septal defect (ASD) in 16 (9.5%), valve regurgitation due to acute rheumatic fever (ARF) in 11 (6.5%), tricuspid valve regurgitation (TR) in six (3.5%) patients, eight (4.7%) patients with bicuspid aortic valve (BAV) and associated regurgitation and stenosis, five (3%) patients with left ventricular hypertrophy (LVH), five (3%) patients with aortic valve regurgitation (AR), two (1.2%) patients, pulmonary valve regurgitation (PR) in one (0.5%) patient, mild pulmonary valve stenosis (PS) in two (1.2%) patients, aortic root dilatation (*z*‐score above 2.5) in three (1.8%) patients, and patent ductus arteriosus (PDA) in one (0.5%) patient (Table 2).

**TABLE 2:** Echocardiographic findings of the cases.

	Female (*n* 73%)	Male (*n* 96%)	Total (*n* 169%)
MR	38 (%52)	47 (%49)	85/50
MVP	17 (%23)	7 (%7.3)	24/14
ASD	6 (%8)	10 (%10.4)	16/9.5
ARF	4 (%5.4)	7 (%7.2)	11/6.5
BAV	0	8 (%8.3)	8/4.7
LV hypertrophy	1 (%1.4)	4 (%4.2)	5/3
TR	2 (%2.7)	4 (%4.2)	6/3.5
AR	1 (%1.4)	4 (%4.2)	5/3
PR	1 (%1.4)	0	1/0.5
LV noncompaction	1 (%1.4)	1 (%1)	2/1.2
PDA	0	1 (%1)	1/0.5
PS	1 (%1.4)	1 (%1)	2/1.2
Aortic dilatation	1 (%1.4)	2 (%2)	3/1.8

Abbreviations: AR, aortic valve regurgitation; ARF, acute rheumatic fever; AS, aortic valve stenosis; ASD, atrial septal defect; BAV, bicuspid aortic valve; LV, left ventriculi; MR, mitral valve regurgitation; MVP, mitral valve prolapses; PDA, patent ductus arteriosus; PR, pulmonary valve regurgitation; PS, pulmonary valve stenosis; TR, tricuspid valve regurgitation.

Twelve (14%) patients with mitral valve regurgitation had moderate regurgitation, and trace regurgitation was detected in other patients. Patients with minimal regurgitation were excluded in this study.

Mitral valve regurgitation was present in 18 (75%) patients with mitral valve prolapse, of whom 14 (58.3%) had mild regurgitation and four (16.6%) had moderate regurgitation. Four patients (16.6%) had isolated MVP.

Patients with left ventricular hypertrophy with interventricular septum systolic thickness ≥ 15 mm and a *Z*‐score ≥ 2.5 were included. Left ventricular outflow tract stenosis was not observed in any of these patients, but was followed up more closely. In the acute rheumatic fever group, three patients (27.3%) had only MR, four patients (28.5%) had only AR, and four patients (28.5%) had both AR and MR. Moderate regurgitation was present in three (27.3%) of these patients, and all of whom received prophylaxis.

For those with secundum ASD, two (12.5%) had a diameter of less than 5 mm, and these patients were followed up. Fourteen (87.5%) patients were referred to an advanced center for ASD closure.

In the group of patients with bicuspid aortic valves, three (37.5%) patients had only AR, one (12.5%) patient had AS, and three (37.5%) patients had both AR and AS. Only one patient (12.5%) had a bicuspid aortic valve, and all of these patients were male. Patients with an aortic root dilatation *z*‐score of 2.5 according to age were included. One patient had a *Z*‐score of 4.2, so they were started on treatment and referred for genetic evaluation. A patient with patent ductus arteriosus was also referred to an advanced center for closure.

Tricuspid valve regurgitation was considered in patients with a jet length over 2.5 m/s. Patients were evaluated in terms of pulmonary hypertension and abnormalities that might cause it. Six patients with tricuspid valve regurgitation and a high right ventricular systolic pressure were referred for otorhinolaryngology. Two of these patients had adenoids that had grown. One patient had a blocked nose due to a severe allergy, and no problems were found in the upper respiratory tract of other three patients. The patients were closely monitored for pulmonary hypertension.

Electrocardiography revealed ventricular extrasystole (VES) in eight (25%) patients, supraventricular extrasystole (SVE) in six (18.7%), ectopic atrial rhythm in five (15.6%), Wolf Parkinson White (WPW) syndrome in four (12.5%), and ST‐T changes in four (12.5) patients. Three (9.4%) patients had Grade 1 atrioventricular node (AV) block (PR over 20 ms), one (3.1%) patient had short PR distance (less than 12 ms), and two (6.3%) patients had right bundle branch block pattern (Table 3).

**TABLE 3:** Electrocardiographic findings of the patients.

ECG	Female (*n*: 17/%)	Man (*n*: 16/%)	Total (33/%)
VES	5/29.4	3/18.8	8/24.2
SVE	3/17.6	3/18.8	6/18.2
Ectopic atrial rhythm	2/11.7	3/18.8	5/15.2
WPW	4/23.5	0	4/12.1
ST‐T change	1/5.8	3/18.8	4/12.1
1st degree AV block	1/5.8	2/12.5	3/9.1
RBBB	1/5.8	1/6.3	2/6.1
Short PR interval	0	1/6.3	1/3

Abbreviations: AV, atrioventricular; RBBB, right bundle branch block; SVE, supraventricular extrasystole; VES, ventricular extrasystole; WPW, Wolf Parkinson White.

In 24‐h rhythm holders performed in patients with ventricular extrasystole, VES ≥ 10% in two patients, VES between 5%–10% in 1one patient, VES between 1%–5% in one patient, and VES below 1% in one patient. Since VES was detected at high heart rates in two patients in the stress tests, treatment was started, and the patient was referred for further examination and treatment. The remaining patients were closely monitored.

Two patients with first‐degree atrioventricular block had acute rheumatic fever (ARF). Patients with Wolf Parkinson White syndrome were referred to the Arrhythmia Center for further examination and treatment after a stress test and 24‐h rhythm Holter monitoring. All patients with Wolf Parkinson's disease female. Three patients (1.5%) had hypertension despite having normal ECHO and ECG results. These patients were referred to the Pediatric Nephrology department. Given that high arterial blood pressure (> 95% P) was detected in the patients' blood pressure Holters', treatment was initiated.

## 4 Discussion

In examinations performed before starting sports, it is necessary to evaluate the health status of individuals in terms of their health status, prediction of abnormalities that may occur during sports activities, the presence of an obstacle to sports, and the physical fitness required for certain sports branches (Corrado, Basso, and Thiene 2005).

Most studies aim to determine the health problems that can lead to sudden cardiac death and how to prevent them.

The European Society of Cardiology (ESC) recommends that athletes should be screened at 12–14 years, which is usually the starting age, and then checked at 2‐year intervals. It is aimed at performing athlete examinations at the beginning of the season to detect disorders in advance and to determine the appropriate road map for the health of the athlete before the competitions begin (Corrado, Pelliccia, et al. 2005; Fritsch et al. 2017) They stated that although methods such as ECG, and ECHO, increase the possibility of diagnosis, they are not recommended because of the cost and impracticality of their application. They stated that these tests could be used for diagnosis and follow‐up in cases where heart disease is suspected (Corrado, Pelliccia, et al. 2005; Maron et al. 2004, 2007).

However, since anamnesis and physical examination alone are insensitive to detecting abnormalities, studies have also shown that the addition of ECG to sports entry examinations increase the sensitivity of screening (Fritsch et al. 2017; Yıldız 2014; Williams et al. 2019).

A study in the USA found that the American Heart Association's 14‐point assessment questionnaire for cardiovascular screening in athletes produced many false positive results with low sensitivity and a low positive predictive value. However, when controlled by experienced clinicians, ECG screening has been shown to outperform the American Heart Association 14‐point test, using all statistical performance criteria (Fritsch et al. 2017).

In addition, unlike Western countries, Japan and Taiwan use and recommend ECG in screening (Niwa et al. 2004; Liu et al. 2020).

When evaluating patients, the presence of complaints such as syncope, chest pain, palpitations, easy fatigue, and dyspnea in the anamnesis should be questioned, and attention should be paid especially to complaints related to effort, and if any, previous surgeries, diseases (Kawasaki Disease, etc.), and chronic medications should also be questioned (Löllgen and Bachl 2015).

Another issue agreed upon in pre‐participation examinations is the detailed examination of the cardiovascular system. Attention should be paid to the phenotypic appearance that indicates a cardiac problem that may accompany the examination (Marfan, Turner, and Ehler‐Danlos syndromes).

In our country, there is no standardized approach for pre‐sports examinations. On the other hand, cardiac evaluation of children and adolescents who want to participate in regular sports activities is performed by creating a strategy based on the practice and experience of the health center or physician.

It has been understood that some of the cases sent to our center came to the pediatric cardiology department because they needed a detailed examination of the heart, while others came to the pediatric cardiology department because the physician or family was extremely concerned about heart diseases. At our hospital, ECG and ECHO examinations were performed after the patient's history and physical examination. However, 24‐h rhythm Holter and stress tests were performed in selected cases.

Çetiner et al. (Çetiner et al. 2019) reported that 19 (11.3%) of 168 children who underwent a pre‐sports examination had abnormal ECHO findings. In a study by Yılmaz et al., 22 (14.3%) of 153 patients had abnormal ECHO results (Yılmaz and Şap 2021).

Çetin et al. (Çetin et al. 2018) evaluated the results of the pre‐sport examination of 380 children aged six and 18 years and reported that nine (2.4%) children had abnormalit*y* in their ECGs. A study on high school children in the USA found abnormal ECGs in 2.8% of the children (Williams et al. 2019).

In our study, 169 patients (8.1%) had abnormal an echocardiogram finding and 33 (1.7%) had abnormal electrocardiograms. In 8 (0.3%) of the patients, there were changes in both ECHO and ECG. The results of our study are in line with those of other studies.

The diseases that are accepted as severe heart problems and recommended to be banned from sports or which may cause sudden death are hypertrophic cardiomyopathy, MVP, aortic valve stenosis, ventricular arrhythmias, supraventricular arrhythmias, WPW, channelopathies, arterial hypertension, aortic rupture, dilated cardiomyopathy, arrhythmogenic right ventricle, and coronary artery diseases (Corrado et al. 2006; Maron et al. 2009).

Our study found that 49 patients (2.3%) had major heart problems. Of these, 24 had mitral valve prolapse (MVP), five had Left Ventricular hypertrophy, three had aortic stenosis due to bicuspid aortic valve, three had aortic dilatation, four had Wolff‐Parkinson‐White (WPW) syndromes, two had LV non‐compaction, two had ventricular extrasystole (VES) at high heart rates, and one had supraventricular tachycardia. In three patients, high blood pressure was also identified.

Although the remaining patients do not have abnormalities that can cause death, it is important because we deal with the pediatric patient population. Because of the high life expectancy of these young patients in age group, they pose a risk in terms of complications that may develop due to these diseases. Diseases such as Atrial Septal Defect (ASD), Mitral Valve Regurgitation (MR), Aortic Valve Regurgitation (AR), and Valve Sequelae due to Acute Rheumatic Fever (ARF), which do not cause major problems in pediatric patients, may gradually increase in valve stenosis or insufficiency during the disease and may cause major valve problems, such as infective endocarditis, or may not be diagnosed when there is a chance for early intervention and can lead to more severe problems.

In addition, patients with a bicuspid aortic valve or associated aortic valve regurgitation have the potential to develop severe, life‐threatening aortic valve stenosis and severe valve regurgitation at a later age. Aortic valve and vascular‐related abnormalities were found more often in males. All patients with bicuspid aortic valves were male.

We believe that it is important to know the patient's medical history and perform a physical examination. This helps us decide which patients should undergo ECG, ECHO, and other advanced heart tests when they are children and want a sports check‐up. However, we think that simply asking about their medical history, performing a physical examination, and asking about their family medical history is not sufficient. We believe that further tests required. While it is important to have sufficient specialists and equipment, and these tests can be expensive, we believe that the potential problems they can cause are too significant to ignore. Therefore, we think that children who play sports should be checked not only for sudden death but also for diseases that could cause major health problems later in life.

Our study shows that approximately one in every 10 patients in the population may have cardiological problems, and 2.3% of these patients have major cardiac problems. Therefore, we recommend that all pediatric patients undergo detailed cardiac evaluation at least once in the early stages of their lives.

### 4.1 Limitations of the Study

As our study was retrospective, detailed anamnesis, family history, and physical examination data were missing and could not be used. Therefore, no comparison could be made regarding which complaints the patients had, what was detected in the family history and physical examination, and what kind of abnormality was detected in which case on ECG and ECHO. This study was conducted in patients with detailed ECHO and ECG data.

## Author Contributions

The authors have made substantial contributions to conception and design, or acquisition of data, or analysis and interpretation of data. Been involved in drafting the manuscript or revising it critically for important intellectual content. Given final approval of the version to be published. All authors should have participated sufficiently in the work to take public responsibility for appropriate portions of the content. Agreed to be accountable for all aspects of the work in ensuring that questions related to the accuracy or integrity of any part of the work are appropriately investigated and resolved.

## Funding

The authors have nothing to report.

## Ethics Statement

This study was prepared according to the Helsinki Declaration. Approval was received from the Turgut Özal University Clinical Research Ethics Committee on 13.05.2024, number 223005.

## Conflicts of Interest

The authors declare no conflicts of interest.

## Data Availability

The data that support the findings of this study are available on request from the corresponding author. The data are not publicly available due to privacy or ethical restrictions.
